# Supplementary material for: Causal Relationship between Aging and Anorexia Nervosa: A White-Matter-Microstructure-Mediated Mendelian Randomization Analysis
Source: Biomedicines. 2024 Aug 16;12(8):1874. doi: 10.3390/biomedicines12081874 (PMC11351342; doi:10.3390/biomedicines12081874)
Supplement: Supplementary file 1 [file biomedicines-12-01874-s001.zip › Supplementary Figure/Figure S4/Singe SNP plot of MR analysis for TL to ubm-b-2027.pdf]

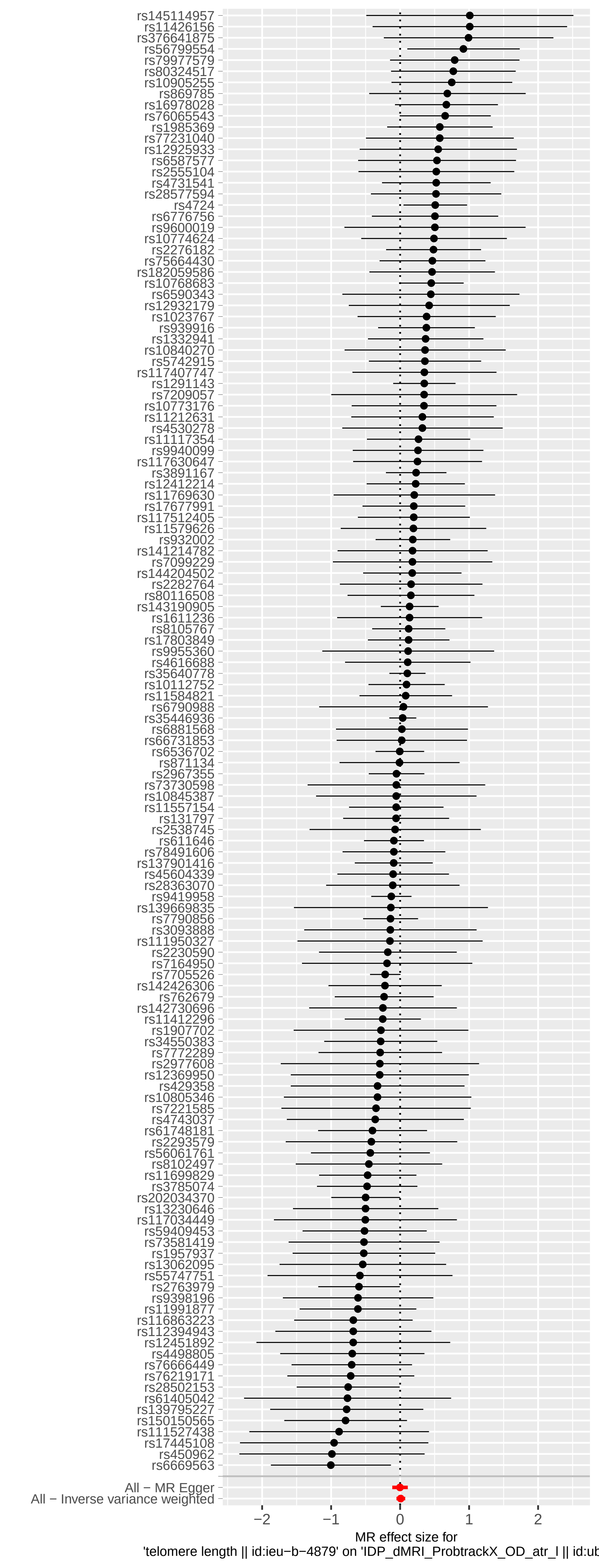
rs145114957
rs11426156
rs376641875
rs56799554
rs79977579
rs80324517
rs10905255
rs869785
rs16978028
rs76065543
rs1985369
rs77231040
rs12925933
rs6587577
rs2555104
rs4731541
rs28577594
rs4724
rs6776756
rs9600019
rs10774624
rs2276182
rs75664430
rs182059586
rs10768683
rs6590343
rs12932179
rs1023767
rs939916
rs1332941
rs10840270
rs5742915
rs117407747
rs1291143
rs7209057
rs10773176
rs11212631
rs4530278
rs11117354
rs9940099
rs117630647
rs3891167
rs12412214
rs11769630
rs17677991
rs117512405
rs11579626
rs932002
rs141214782
rs7099229
rs144204502
rs2282764
rs80116508
rs143190905
rs1611236
rs8105767
rs17803849
rs9955360
rs4616688
rs35640778
rs10112752
rs11584821
rs6790988
rs35446936
rs6881568
rs66731853
rs6536702
rs871134
rs2967355
rs73730598
rs10845387
rs11557154
rs131797
rs2538745
rs611646
rs78491606
rs137901416
rs45604339
rs28363070
rs9419958
rs139669835
rs7790856
rs3093888
rs111950327
rs2230590
rs7164950
rs7705526
rs142426306
rs762679
rs142730696
rs11412296
rs1907702
rs34550383
rs7772289
rs2977608
rs12369950
rs429358
rs10805346
rs7221585
rs4743037
rs61748181
rs2293579
rs56061761
rs8102497
rs11699829
rs3785074
rs202034370
rs13230646
rs117034449
rs59409453
rs73581419
rs1957937
rs13062095
rs55747751
rs2763979
rs9398196
rs11991877
rs116863223
rs112394943
rs12451892
rs4498805
rs76666449
rs76219171
rs28502153
rs61405042
rs139795227
rs150150565
rs111527438
rs17445108
rs450962
rs6669563
All − MR Egger
All − Inverse variance weighted
−2
−1
0
1
2
MR effect size for
'telomere length || id:ieu−b−4879' on 'IDP_dMRI_ProbtrackX_OD_atr_l || id:ub
